# Aseptic Meningitis with Urinary Retention: A Case Report

**DOI:** 10.1155/2011/741621

**Published:** 2011-10-30

**Authors:** Fotinie Ntziora, Aristidis Alevizopoulos, Kostas Konstantopoulos, Sofia Kanellopoulou, Dimitrios Bougas, Konstantinos Stravodimos

**Affiliations:** ^1^1st Academic Department of Medicine, Laiko General Hospital, Medical School, University of Athens, 11527 Athens, Greece; ^2^1st Academic Department of Urology, Laiko General Hospital, Medical School, University of Athens, 11527 Athens, Greece

## Abstract

*Introduction*. Aseptic meningitis is serious inflammation of the meninges caused by agents including viruses, non-viral pathogens, non-infectious conditions and chemicals. *Case Presentation*. This study concerns the case of a 16-year-old healthy Greek female with persistent fever, mild headache and acute urinary retention, secondary to aseptic meningitis. Physical examination revealed no distinct signs of meningeal irritation. The urinary bladder was palpable, painless and over-distended. Serology carried out for common viruses was as follows: CMV IgG (−), CMV IgM (−), HSV IgG (−), HSV IgM (+), VZ IgG (+), VZ IgM (−), EBV IgG (−) and EBV IgM (+). During recovery in hospital, three trials of removing a urinary catheter were carried out; during the first two attempts the patient was unable to urinate and had a loss of bladder sensation. On the third attempt the patient had modest bladder perception but she left a post-voiding residual, and was instructed to perform bladder self-catheterization. Seven days after being discharged the patient underwent a full recovery. *Conclusion*. There are few reports concerning aseptic meningitis together with acute urinary retention. A number of these cases concern so-called “meningitis-retention syndrome,” which implies an underlying CNS mechanism, while others concerned an underlying peripheral nervous system mechanism.

## 1. Introduction

Aseptic meningitis is serious inflammation of the meninges caused by non-bacterial agents including viruses, non-viral pathogens, and non-infectious conditions such as systemic lupus erythematosus, leukemia, lymphoma, and nonsteroidal anti-inflammatory drugs (NSAIDs) and other chemicals. Enteroviruses are responsible for more than 90% of aseptic meningitis cases, particularly during summer and autumn. However, other viruses including flavivirus, herpesvirus, and mumps can cause meningitis [1]. In the majority of cases, patients are admitted with fever, headache, a stiff neck, nausea, and vomiting. However, patients can present with a variety of symptoms ranging from asymptomatic pleocytosis in the cerebrospinal fluid (CSF) to a serious neurological deficiency. 

This paper presents the case of a young female patient with acute urinary retention secondary to aseptic meningitis. The combination of urinary retention and meningitis is described in the literature, but it is considered uncommon.

## 2. Case Presentation

A 16-year-old healthy Greek female was admitted to hospital for seven days due to persistent fever, mild headache, and acute urinary retention. During this time, the patient complained of urinary retention and was subjected to catheterization. She reported a rash on the front of the thorax that had receded by the time she arrived at our department. She had been treated with azithromycin for one day, followed by ceftriaxone for seven days before being admitted to us. At the time of discharge, the patient was afebrile, but unable to urinate in three trials where a catheter was removed. Therefore, the patient was discharged from our department in possession of a Foley catheter. Her past medical history was unremarkable; no smoking, drinking, or sexual activity was reported.

On admission, the patient was febrile, with a body temperature of 37.8°C. Physical examination revealed no distinct findings or signs of meningeal irritation. The patient had an ail guise with dry mouth mucosa, with no mucocutaneous lesions and complained of nausea and vomiting. On auscultation, no additional respiratory sounds were noted; the liver was slightly palpable. The urinary bladder was palpable, painless, and overdistended; urinary catheterization yielded 500 cc of urine. The physical examination was otherwise normal.

Hematology was as follows: haematocrit 39%, haemoglobin 12.4 g/dL, white blood cells 7970/mm^3^ (39% polymorphonuclears, 43% activated lymphocytes, 13% monocytes, and 3% atypical cells), and platelets 320000/mm^3^. C-reactive protein was normal, and the erythrocyte sedimentation rate was 54 mm/h during the 1st hour. Serum biochemistry was normal with the exception of a minimal elevation in *γ*GT (112 IU/L), ALT (72 IU/L) and LDH (572 IU/L).

In view of the presence of fever, headache, and acute urinary retention, a lumbar puncture was performed on admittance. The CSF cells were recorded as 100/mm^3^ lymphocytes, protein was elevated (51 mg/dL), and glucose was reduced (46 mg/dL), with serum glucose at 93 mg/dL. Gram staining of the CSF was negative for microorganisms, and an India ink test was negative for *Cryptococcus*.

CSF cultures, blood cultures, and urine cultures were negative. Serology for cytomegalovirus (CMV), herpes virus (HSV), varicella zoster virus (VZ), and Epstein-Barr virus (EBV) was as follows: CMV IgG (−), CMV IgM (−), HSV IgG (−), HSV IgM (+), VZ IgG (+), VZ IgM (−), EBV IgG (−), and EBV IgM (+). CSF polymerase chain reaction (PCR) for CMV was negative. Serum was negative for *Borrelia burgdorferi* and *Listeria monocytogenes* antibodies. A Wright test was negative, as was a tuberculin skin test.

Fever and urinary retention persisted, and a stiff neck was evident. Therefore, lumbar puncture was repeated on day 14 after the onset of symptoms (the 6th day of hospitalization in our department). In the CSF, there were 20/mm^3^ lymphocytes, 45 mg/dL glucose (with serum glucose 93 mg/dL), and 52 mg/dL protein. Gram staining of the CSF was negative for bacteria, and an ink test for *Cryptococcus* was negative (Table 1).

A chest X-ray revealed no abnormalities, and abdominal ultrasonography revealed no abnormalities with the exception of a slight enlargement of the liver (122 mm); heart ultrasonography was normal. After the second lumbar puncture was performed (on the 6th day of hospitalization and 14 days after the onset of symptoms), brain magnetic resonance imaging (MRI) was performed but no lesion associated with the urinary retention was detected. Owing to the persistence of symptoms, a lumbar spinal cord MRI was performed a week later; this was negative.

On admittance, the patient continued antimicrobial treatment with ceftriaxone; acyclovir was administered, and following the first lumbar puncture ampicillin was administered. After the second lumbar puncture was performed, gentamycin was administered for three days. 

The patient remained afebrile eight days after being admitted to our department. Her general clinical condition and stiff neck had improved, but urinary retention persisted. During hospital recovery, three trials concerning removing the urinary catheter were performed; the first on day three, without additional treatment, the second and third on days 10 and 15, respectively, under treatment with an *α*-1 blocker (tamsulosin 0.4 mg, once a day). During the first two attempts, the patient was unable to urinate and she presented with a complete loss of bladder sensation. On the third attempt, she had modest bladder perception and was able to urinate, but she left a postvoiding residual of 450 mL and had to be catheterized. The patient was instructed to perform bladder self-catheterization and, seven days after being discharged, she was able to urinate by herself. She was advised to perform an urodynamic control but was lost to followup.

## 3. Discussion

This paper presents the case of a young female patient referred to our department due to persistence of fever and unresolved urinary retention. The clinical symptoms such as fever, headache, and stiff neck are typical manifestations of meningitis. Furthermore, the lymphocyte cell types in the CSF analysis suggested aseptic meningitis rather than bacterial. Urinary retention, secondary to meningitis, is rarely encountered but has been described in the literature. The majority of cases have been attributed to viruses; the exceptions are one case caused by Listeria [2] and two cases of meningococcal meningitis [3, 4]. 

Herpes virus is the most common cause of meningitis associated with urinary retention [5–8]. The patient was positive for IgM EBV, indicating that this could have been the cause of the aseptic meningitis. The most probable mechanism of urinary retention in such cases is the direct involvement of pelvic nerves, with subsequent detrusor areflexia [9, 10]. A generic sacral or subsacral lesion can lead to an acontractile detrusor, incompetent urethra, and loss of bladder sensation. Viral infection of the sacral roots, predominantly attributed to HSV infection, can lead to reversible urinary retention by causing localized lumbosacral meningomyelitis or infectious neuritis of the pelvic nerves [11]. It is likely that this was the mechanism underlying the retention in this case, although an urodynamic control was not performed.

In contrast to other cases reported, the patient presented with a stiff neck during the course of the illness and not at the onset. Symptoms associated with meningitis such as disturbance of consciousness, hypesthesia, weakness and disturbed reflexes of the lower extremities, fecal incontinence, tetraparesis, and diplopia were absent. Therefore, the bladder dysfunction could not be attributed to a consciousness level disturbance [12, 13]. It could be suggested that the preexisting EBV infection was associated with polyradiculoneuritis, which could explain the coexistence of meningitis and urinary retention. The pathogenesis is uncertain; direct infection and parainfectious mechanisms could have played a role. Various hypotheses regarding the mechanisms underlying viral transportation from the circulatory system to the nervous system have been suggested recently and include moving through damaged endothelium, direct infection of the endothelial cells, and ligation through migrating leukocytes. As in other cases, the patient was previously healthy, and developed acute urinary retention six days after the onset of symptoms relating to meningitis, while urinary retention was resolved only after complete recovery from the meningitis symptoms [14, 15].

## 4. Conclusion

There are few reports concerning aseptic meningitis with acute urinary retention. Several of such cases concern meningitis-retention syndrome, which affects the central nervous system, while others include an underlying peripheral nerve system mechanism. It appears that the present case belongs to the latter group, with a transient viral lesion of the sacral nerves being involved in the urinary retention. Further investigation is required to elucidate the exact mechanism underlying the retention and for optimum treatment of such cases.

## Figures and Tables

**Table 1:** Comparison of the lumbar puncture results.

	1st LP	2nd LP
Cells (/mm^3^)	100	20
Glucose (mg/dL)	46	45
Protein (mg/dL)	51	52
LDH (U/L)	42	61
Cl^−^ (mEq/L)	120	119
Gram stain	Negative	Negative
*Cryptococcus*	Negative	Negative
PCR CMV	Negative	—
